# Odorant degrading enzyme candidates enriched in the antennae of the white-spotted flower chafer, *Protaetia brevitarsis*

**DOI:** 10.3389/finsc.2026.1843786

**Published:** 2026-06-03

**Authors:** Yi Lu, Peng Gao, Xuan Wang, Junwei Yuan, Haie Zhang, Jingzheng Zhang

**Affiliations:** 1 Engineering Research Center of Chestnut Industry Technology, Ministry of Education, Hebei Normal University of Science and Technology, Qinhuangdao, Hebei, China; 2 College of Agronomy and Biotechnology, Hebei Normal University of Science and Technology, Changli, Hebei, China

**Keywords:** carboxylesterases, cytochrome P450s, glutathione S-transferases, odorant degrading enzymes, *Protaetia brevitarsis*, UDP-glycosyltransferases

## Abstract

The white-spotted flower chafer (*Protaetia brevitarsis*) exhibits a preference for feeding on mature fruits of various crops, making it a significant agricultural pest. Pesticide application often results in fruit residues and environmental contamination, highlighting the need for eco-friendly and highly effective alternative control strategies. For instance, targeted regulation of the pest’s olfactory system could be used to influence its behavior. This study focused on the enzymes responsible for terminating olfactory signals, namely odorant degrading enzymes (ODEs). Through antennal transcriptome sequencing, four related gene families comprising a total of 94 genes were identified, including 23 carboxylesterases, 40 cytochrome P450s, 21 glutathione S-transferases, and 10 UDP-glycosyltransferases. The majority of enzyme proteins possessed typical domain characteristics and key residues, suggesting their potential catalytic activity. These enzymes clustered into multiple clades in the phylogenetic tree, likely undertaking diverse physiological functions including odor substance metabolism. To more precisely determine the ODE candidates, tissue expression profiling was conducted on 17 highly expressed genes (FPKM >100). The results revealed that 16 genes were enriched or specifically expressed in the antennae. We propose these genes as primary candidates for ODEs, serving as a prioritized list for future functional validation. Our work lays the foundation for elucidating the odor signal inactivation mechanism in *P. brevitarsis*, expands the transcriptomic resource of ODEs in Scarabaeidae, and provides a theoretical basis for developing novel pest management targets based on olfactory behavior.

## 1 Introduction

The white-spotted flower chafer, *Protaetia brevitarsis* (Coleoptera: Scarabaeidae), is widely distributed in East Asian countries and the Russian Far East (1). The adults are characterized by a long developmental period and a polyphagous diet, particularly favoring the mature fruits of crops such as grapes, peaches, corn, and tomatoes. This behavior leads to severe yield reduction and substantial quality decline in crops, making them a highly threatening pest in agricultural production (2). Traditional chemical control methods are prone to causing pesticide residues in fruits and environmental pollution. Meanwhile, the adults possess strong flight capability and a hard exoskeleton, which compromises chemical control efficacy (3). Given the pronounced chemotactic and aggregation feeding behaviors of *P. brevitarsis* adults (4, 5), elucidating the molecular mechanisms underlying the olfactory recognition of *P. brevitarsis* and identifying potential targets for green pest control represent highly valuable research directions in this field. Zhao et al. identified six classes of olfactory genes from the antennae of *P. brevitarsis*, including 66 odorant receptors, 20 ionotropic receptors, 10 gustatory receptors, 13 odorant-binding proteins, 4 chemosensory proteins, and 4 sensory neuron membrane proteins (6). In the same year, Liu et al. discovered that three antennal-specific odorant-binding proteins were involved in recognition of common floral volatiles such as β-ionone, phenethyl salicylate, phenylacetaldehyde, and benzyl benzoate (7). However, there have been no reported studies on odorant degrading enzymes (ODEs) in the white-spotted flower chafer to date.

During the olfactory recognition process of insects, once odor molecules activate the receptors, they must be rapidly inactivated by ODEs to avoid signal saturation interference, thereby restoring the sensitivity of olfactory sensory neurons and enabling insects to continuously detect new odorants in the environment (8). ODEs are a collective term for enzymes capable of metabolizing odor molecules. Based on the differences in their functional stages, they can be classified into two categories. The first category primarily includes carboxylesterases (CXEs) and cytochrome P450s (CYPs), which convert hydrophobic odor molecules into intermediate products containing polar groups (such as carboxyl and hydroxyl groups). The second category mainly consists of glutathione S-transferases (GSTs) and UDP-glycosyltransferases (UGTs), which catalyze the binding of these intermediates with endogenous substances to generate highly water-soluble compounds for excretion.

CXEs belong to a subgroup of serine esterases, which specifically catalyze the hydrolysis of substrates containing ester bonds (9, 10). A diverse array of CXEs in insects are involved in various physiological processes, including hormone/pheromone processing, detoxification of xenobiotics, and neurodevelopment (11). CXEs related to odor degradation have been successively reported in *Antheraea polyphemus* and *Popilia japonica*. Since these enzymes can rapidly inactivate ester sex pheromone components, they have been classified as pheromone degrading enzymes (PDEs) (12–14). Considering that esters are the main components of sex pheromones in Lepidoptera, multiple kinetic studies have been conducted on CXEs in moth species. Research findings indicated that CXEs enriched in antennae participated in the degradation of sex pheromones and/or plant volatile esters (15–17).

CYPs are also a multifunctional family of metabolic enzymes capable of catalyzing various reactions such as oxidation, reduction, and hydroxylation (18). Early studies have demonstrated that specific CYP in *Phyllopertha diversa* possess pheromone-degrading activity, indicating their role in olfactory signal inactivation (19, 20). In the tobacco cutworm *Spodoptera litura*, electroantennogram responses of adults to two sex pheromone components were significantly attenuated following gene knockdown of *SlituCYP4L4* (21). In the red imported fire ant (*Solenopsis invicta*), silencing either *SinvCYP6K1* or *SinvCYP4V2* impaired its recognition of alarm pheromones (22). Other study has shown that an antenna-specific CYP from the mountain pine beetle can metabolize monoterpene volatiles in plants (23).

The core function of GSTs is to catalyze the conjugation of electrophilic substrates with reducing glutathione (24). GSTs expressed in chemical sensory organs are believed to be involved in odor clearance (24). For example, GST-msolf1, specifically expressed in the antennae of the sphinx moth *Manduca sexta*, was located in olfactory sensilla sensitive to sex pheromones and could inactivate aldehyde odorants (25). In recent years, Assays of recombinant enzyme activity have been conducted on GSTs highly expressed in the antennae of various insects, revealing their degradation activity toward multiple volatile substrates such as alcohols, aldehydes, and esters (26–28). Furthermore, in *Holotrichia parallela*, a metabolite of cinnamaldehyde (one of the aldehyde substrates) was identified via high-resolution mass spectrometry, with a molecular weight (439) equal to the sum of glutathione (307) and cinnamaldehyde (132) (28).

UGTs play a crucial role in the metabolism of lipophilic substances by enhancing their water solubility through glycosylation (29, 30). High expression of UGT genes has been detected in the antennae of various insects, suggesting their potential involvement in olfaction (31–33). Moreover, mutations and RNA interference studies have demonstrated that UGT36E1, expressed in olfactory sensory neurons of *Drosophila melanogaster*, participates in the detection of sex pheromones (34). Additionally, *UGT46A1* in *Bombyx mori* might influence feeding behavior by mediating olfactory sensitivity (35).

Although these enzymes perform complex physiological functions, members enriched in antennae are generally considered associated with odor degradation (8). In this study, we systematically analyzed four major classes of enzymes related to odor degradation from the antennal transcriptome of *P. brevitarsis*. By integrating phylogenetic analysis with tissue expression profiling, we precisely identified antennae-enriched candidates to generate a reliable prioritized list. This comprehensive dataset not only enriches the molecular understanding of ODEs in Scarabaeidae but also lays a solid foundation for elucidating the mechanisms of odor signal inactivation in *P. brevitarsis*.

## 2 Materials and methods

### 2.1 Insects and tissue collection

Adult *P. brevitarsis* used in this study were purchased from a grubs breeding base in Fuyang City, Anhui Province, China. The larvae were reared in fully decomposed agricultural organic waste, and newly emerged adults were fed with fruits. Healthy seven-day-old adults were selected for tissue sample collection. The sexes were distinguished by dissecting the external genitalia of adults, followed by isolation of antennae, head, leg, and abdominal tissues from male and female adults respectively. Total RNA was extracted individually from male antennae (40 pieces) and female antennae (40 pieces) for transcriptome sequencing, with RNA-seq analysis conducted in three biological replicates. Other tissue samples were mixed at a 1:1 sex ratio before RNA extraction, specifically head mixtures (2 heads per sex), leg mixtures (3 legs per sex), and abdominal mixtures (1 abdomen per sex). All samples were cryopreserved at −80 °C.

### 2.2 RNA extraction, cDNA library construction and sequencing

Total RNA was extracted from tissue samples using the RNApure Total RNA Kit (Aidlab, Beijing, China). RNA concentration and purity were determined by the NanoDrop One spectrophotometer (Thermo Fisher Scientific, USA). RNA integrity was assessed by agarose gel electrophoresis, and RQN values were measured using the Agilent 5300 Fragment Analyzer. The qualified total RNA from female and male antennae was then submitted to Shanghai Major Biomedical Technology Co., Ltd. (Shanghai, China) for cDNA library construction and sequencing.

The brief workflow for cDNA library construction is as follows. First, mRNA was isolated from total RNA using Oligo(dT)-biotinylated magnetic beads. Fragmentation buffer was then added to cleave the mRNA into fragments approximately 300bp in length. Using random primers and reverse transcriptase, the first strand of cDNA was synthesized from the mRNA template. Subsequently, the second-strand cDNA was synthesized. Next, the double-stranded cDNA underwent end-repair and 3’-end adenylation. Adapter sequences were then ligated, and the fragments were screened for size before PCR amplification. Finally, the qualified library was sequenced on the NovaSeq X Plus platform.

### 2.3 *De novo* assembly and functional annotation

The raw reads obtained from sequencing were filtered to remove low-quality reads and adapters, thereby obtaining clean reads. Trinity software (version 2.8.5) was used to perform *de novo* assembly of all clean data (36). The assembled sequences were filtered and optimized using TransRate (version 1.0.3) (37) and CD-HIT (version 4.5.7) (38). The integrity of transcriptome assembly was evaluated using BUSCO (version 3.0.2) (39).

To obtain comprehensive annotation information, all unigenes were searched against six public databases (NCBI NR (v2023.07), Swiss-Prot (v2023.11), Pfam (v36.0), EggNOG (v2020.06), Gene Ontology (GO), and KEGG (v2023.09)) using the BLASTx algorithm (implemented in BLAST+ v2.9.0) with an E-value cutoff of 1e-5. For functional annotation, the best hit with the lowest E-value and highest sequence identity (for NR/Swiss-Prot) was retained for each unigene. The expression levels of unigenes were quantitatively analyzed using the RSEM software (version 1.3.1) (40), calculated as FPKM (fragments per kilobase per million mapped fragments) (41).

### 2.4 Genes identification and sequences analysis

According to functional annotation results of the antennae transcriptomes, candidate genes encoding CXEs, CYPs, GSTs, and UGTs were preliminarily screened through keyword searches. These were then validated by querying the NCBI non-redundant protein sequence database with BLASTx (https://blast.ncbi.nlm.nih.gov/Blast.cgi, accessed on 10 October 2025). Successful validated genes were further processed using ORF Finder (https://www.ncbi.nlm.nih.gov/orffinder/, accessed on 10 October 2025) to identify open reading frames (ORFs) and generate translation results. Protein molecular weight (Mw) and isoelectric point (pI) were calculated via Expasy (https://web.expasy.org/compute_pi/, accessed on 13 October 2025). The SignalP 6.0 (https://services.healthtech.dtu.dk/services/SignalP-6.0/, accessed on 13 October 2025) was employed to predict signal peptides for CXEs, GSTs, and UGTs, while the DeepTMHMM 1.0.44 (https://dtu.biolib.com/DeepTMHMM/, accessed on 13 October 2025) was utilized to predict transmembrane domains for CYPs and UGTs. Conserved domains and catalytic residues were identified using the NCBI Conserved Domain Search (https://www.ncbi.nlm.nih.gov/Structure/cdd/wrpsb.cgi, accessed on 14 October 2025) to confirm the structural basis of gene family classification.

### 2.5 Phylogenetic analysis

Phylogenetic analyses were conducted separately on the identified CXEs, CYPs, GSTs, and UGTs of *P. brevitarsis*. The species included in the analysis comprised model insects such as *D. melanogaster*, *B. mori*, *Apis mellifera*, and *Tribolium castaneum*; Scarabaeidae species including *H. parallela*, *Holotrichia oblita*, *Popillia japonica*, *Onthophagus taurus*, *Oryctes borbonicus*, and *Trypoxylus dichotomus*; as well as other Coleoptera species, such as *Tenebrio molitor*, *Leptinotarsa decemlineata*, *Dendroctonus ponderosae*, *Rhaphuma horsfieldi*, and *Lissorhoptrus oryzophilus*, etc. The accession numbers of sequences used for phylogenetic analyses are detailed in **Supplementary Table 1** (42–46).

Amino acid sequences were aligned with MAFFT (version 7.505) (47), followed by removing of the gap sites with trimAl (version 1.2rev57) (48). Maximum likelihood phylogenies were inferred using IQ-TREE (version 3.0.1) (49) under the model automatically selected by IQ-TREE for 1000 ultrafast (50) bootstraps. Finally, the phylogenetic trees were visualized using the iTOL online editor (https://itol.embl.de/, accessed on 16 October 2025).

### 2.6 Tissue expression profile by RT-qPCR

To validate high-abundance core ODE candidates, an FPKM >100 threshold was applied based on the principle of predominant expression in olfactory tissues (8), thereby minimizing background noise. Reverse transcription-quantitative PCR (RT-qPCR) was then performed to compare the relative expression levels of these selected genes among male antennae, female antennae, head, leg, and abdomen.

For this analysis, total RNA extracted in Section 2.2 was reverse-transcribed into first-strand cDNA using the PrimeScript™ RT reagent Kit with gDNA Eraser (Takara, Dalian, China). Specific primers for the target genes and the reference gene (*Ribosomal Protein L10*, *RPL1*0) were designed using the transcriptome sequences (**Supplementary Table 2**).

qPCR was performed according to the instructions of TB Green *Premix Ex Taq* II (Takara, Dalian, China). The experiment was set up with a no-template control (NTC) and three technical replicates. Primer specificity was evaluated by melting curve analysis. The qPCR reaction mixture (20 μL) consisted of: 10 μL of TB Green *Premix Ex Taq* II, 0.8 μL (10 pmol/μL) of each primer, 4.8 μL (8 ng) of cDNA template, and 3.6 μL of sterilized ultrapure water. The reaction was conducted on the CFX96 Real-Time PCR Detection System (Bio-Rad, USA) with the following program settings: 95 °C for 30 s; 95 °C for 5 s, 60 °C for 30 s, for 40 cycles.

Relative expression levels were calculated using the 2^-ΔΔCt^ method (51). Statistical analysis was performed with SPSS (version 27.0) software, employing one-way ANOVA to analyze expression differences among tissues and Tukey’s test (*p* < 0.05) for multiple comparisons. Expression profiles were plotted using Origin 2024.

## 3 Results

### 3.1 Sequence assembly and functional annotation

The raw data obtained from antennal transcriptome sequencing of male and female *P. brevitarsis* were deposited in the NCBI Sequence Read Archive (SRA) database with accession number SRR37408762 and SRR37408763. After filtering, 43.01 Gb of clean data was obtained, with each sample’s clean data exceeding 6.11 Gb and the percentage of Q30 bases consistently above 95.9%. Assembly resulted in 100,015 transcripts and 58,243 unigenes with a N50 length of 1,619 bp (**Table 1**). The BUSCO score (C: 96.2%[S: 92.6%; D: 3.6%]) indicated good integrity of the transcriptome assembly (**Table 1**).

**Table 1:** Optimization assembly results.

Type	Unigene	Transcript
Total number	58243	100015
Total base	50382404	95783124
Largest length (bp)	15616	15616
Smallest length (bp)	201	201
Average length (bp)	865.04	957.69
N50 length (bp)	1619	1796
E90N50 length (bp)	2942	2675
Fragment mapped percent (%)	71.676	82.377
GC percent (%)	36.71	36.21
TransRate score	0.34836	0.4059
BUSCO score	C: 96.2%[S: 92.6%; D: 3.6%]	C: 98.2%[S: 65.5%; D: 32.7%]

The number of unigenes with functional annotation in six databases (NR, Swiss-Prot, Pfam, EggCOG, GO, and KEGG) is shown in **Supplementary Figure 1A**. Among these, 24,450 (41.98%) unigenes were matched in the NCBI NR database. Species distribution revealed that over half of the annotations originated from three species: *H. oblita*, *O. borbonicus*, and *O. taurus* (**Supplementary Figure 1B**). Using the GO database, 18,755 (32.2%) unigenes were assigned biological terms. At the Level 2 hierarchy, the primary functional categories included “cellular process”, “metabolic process”, “cell part”, “binding”, and “catalytic activity” (**Supplementary Figure 1C**). Additionally, 14,832 (25.47%) unigenes were annotated in the KEGG database, with metabolic pathways categorized into six classes: “Metabolism”, “Genetic Information Processing”, “Environmental Information Processing”, “Cellular Processes”, “Organismal Systems”, and “Human Diseases” (**Supplementary Figure 1D**).

### 3.2 Identification of *CXEs*

A total of 23 *CXEs* were identified, numbered as *PbreCXE1-PbreCXE23* (**Supplementary Table 3**). Among them, 20 *PbreCXEs* possessed complete ORFs encoding 540–746 amino acids. Signal peptides were predicted at the N-terminus of 18 PbreCXEs (**Supplementary Table 3**). Blastx alignment results demonstrated that these proteins shared 54%-88% sequence identity with CXEs from closely related species (**Supplementary Table 3**). Through multiple sequence alignment and conserved domains analysis, it was found that most PbreCXEs exhibited typical features such as the oxyanion hole, a pentapeptide motif of Gly-X-Ser-X-Gly (“X” represents any residue) o), and a catalytic triad of Ser-Glu-His. In contrast, PbreCXE21 lacked Ser and His, PbreCXE23 lacked Glu and His, and PbreCXE22 was deficient in all catalytic residues, suggesting that these three proteins may lack catalytic activity (**Supplementary Figure 2**).

**Figure 1:**
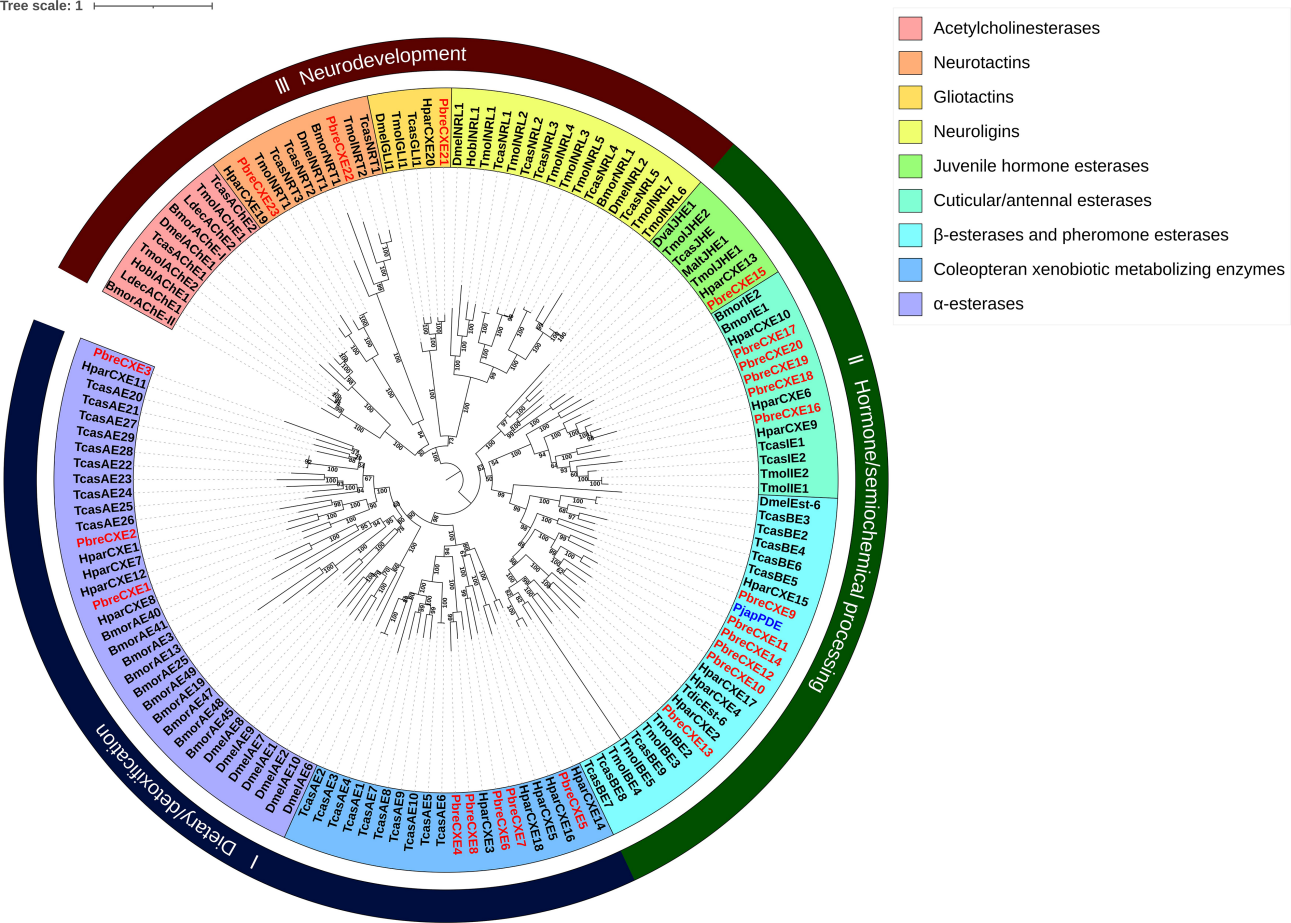
Phylogenetic analysis of CXEs from *P. brevitarsis* (Pbre) and other insects including *D. melanogaster* (Dmel), *B. mori* (Bmor), *T. castaneum* (Tcas), *H. parallela* (Hpar), *T. molitor* (Tmol), *L. decemlineata* (Ldec), *H. oblita* (Hobl), *P. japonica* (Pjap), *T. dichotomus* (Tdic), *Dendroctonus valens* (Dval), and *Monochamus alternatus* (Malt). Accession numbers of the sequences or references to their sources are listed in **Supplementary Table 1**. The PbreCXEs are highlighted in red.

According to the classification system proposed by Oakeshott et al., the phylogenetic tree was divided into three functional groups: I Dietary/detoxification (8 PbreCXEs), II Hormone/semiochemical processing (12 PbreCXEs), and III Neurodevelopmental (3 PbreCXEs) (11) (**Figure 1**). Each functional group was subdivided into 2–3 clades (**Figure 1**). Most enzymes in Groups I and II possessed catalytic functions, including clades related to metabolism of xenobiotics and pheromone processing, such as “Coleopteran xenobiotic metabolizing enzymes”, “β-esterases and pheromone esterases”, and “Cuticular/antennal esterases” (**Figure 1**). Notably, four PbreCXEs with high expression levels (FPKM >100) in male or female antennae were classified into these clades (**Supplementary Table 3**). Besides, PbreCXE21 and PbreCXE23 belonged to the non-catalytic Group III enzymes, specifically the “Gliotactins” and “Neurotactins” clades (**Figure 1**), which is consistent with the results of the conserved domain analysis.

### 3.3 Identification of *CYPs*

Forty *PbreCYPs* were identified, all possessing intact ORFs with coding lengths ranging from 470 to 554 amino acids (**Supplementary Table 3**). These sequences have been submitted to the Committee on Standardized Cytochrome P450 Nomenclature for naming, as detailed in **Supplementary Table 3**. The predicted molecular weights ranged from 54 to 63 kDa, with isoelectric points of 6.40 to 9.31 (**Supplementary Table 3**). 25 PbreCYPs were predicted to contain N-terminal transmembrane domains (**Supplementary Table 3**). Based on the best Blastx matches, the sequence identity of CYPs in *P. brevitarsis* and other species of the same family exceeded 47% (**Supplementary Table 3**). Multiple sequence alignment and analysis indicated that all PbreCYPs sequentially contained the following conserved domains from the N-terminus to the C-terminus: C-helix, I-helix, K-helix, PERF motif, and Heme-binding motif (**Supplementary Figure 3**).

**Figure 2:**
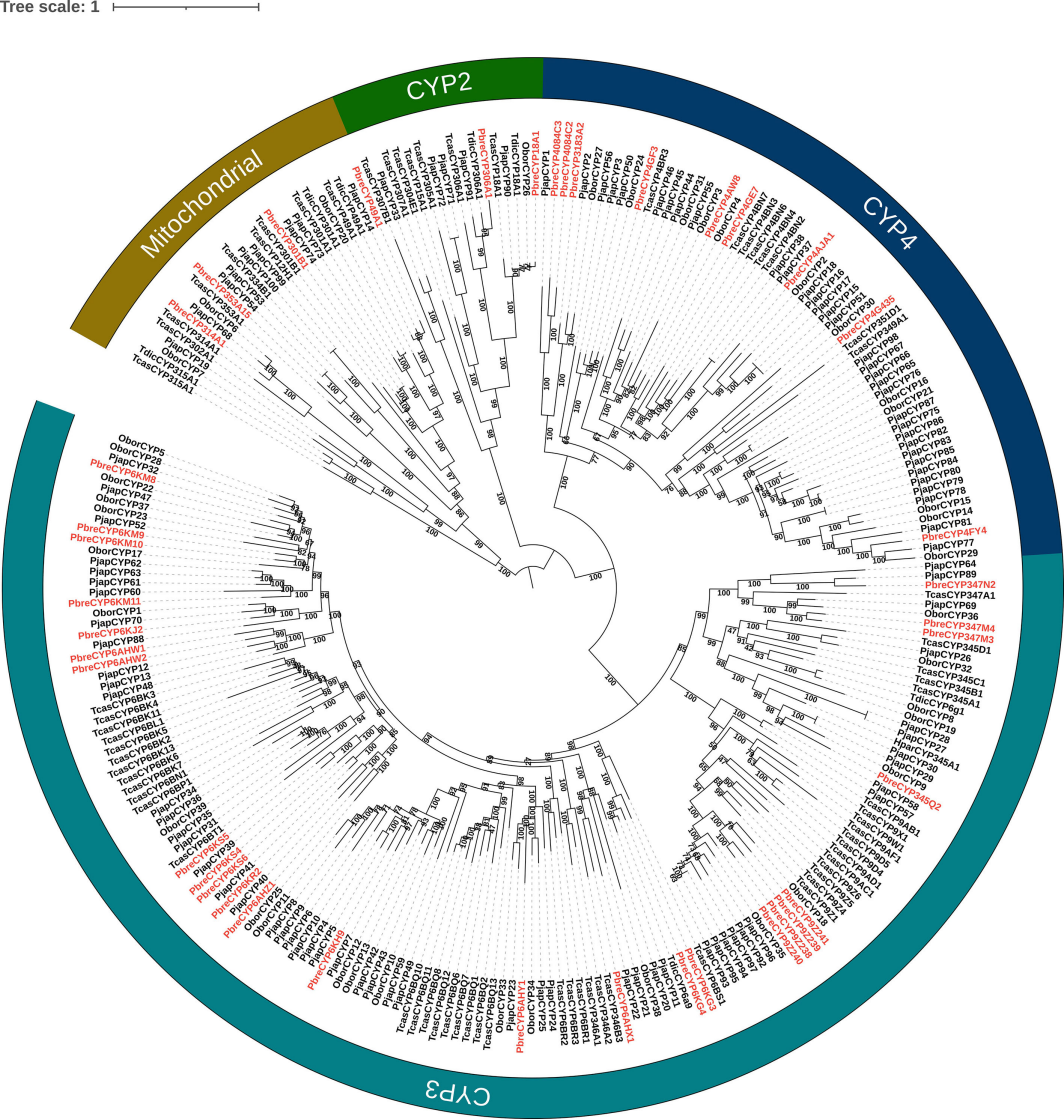
Phylogenetic analysis of CYPs from *P. brevitarsis* (Pbre) and other insects including *T. castaneum* (Tcas), *O. borbonicus* (Obor), *P. japonica* (Pjap), *H. parallela* (Hpar), and *T. dichotomus* (Tdic). Accession numbers of the sequences or references to their sources are listed in **Supplementary Table 1**. The PbreCYPs are highlighted in red.

As illustrated by the phylogenetic tree (**Figure 2**), these CYPs were classified into four clans—CYP2, CYP3, CYP4, and Mitochondrial—which were further subdivided into 13 families (**Supplementary Table 3**). Similar to the observations in *T. castaneum*, CYP3 and CYP4 exhibited significant gene amplification (52). Particularly, families 6, 4, and 9 contained more than half of the total PbreCYP members, with 17,6, and 4 members respectively (**Figure 2**). Five highly abundant genes—*PbreCYP4AW8*, *PbreCYP6KM9*, *PbreCYP6AHW1*, *PbreCYP9Z240*, and *PbreCYP347N2*—were identified in these two clans, with FPKM values ranging from 152 to 921 (**Supplementary Table 3**).

### 3.4 Identification of *GSTs*

Twenty-one genes encoding GSTs were identified, including 20 cytosolic GSTs and 1 microsomal GST, none of which had predicted signal peptides at their N-terminus (**Supplementary Table 3**). Except for *PbreGSTe2* and *PbreGSTs2*, all other *GSTs* possessed intact ORFs encoding 150–249 amino acids (**Supplementary Table 3**). These proteins exhibited sequence identity of 51%-89% with those from other Coleoptera insects (**Supplementary Table 3**). Conserved domain predictions revealed that 14 GSTs had a GSH-binding site (G-site) at their N-terminus, while 16 GSTs possessed a substrate-binding pocket (H-site) at their C-terminus (**Supplementary Figure 4**). Sequence alignment of PbreMGST1 with eight microsomal GSTs from five insect species revealed a highly conserved motif and four transmembrane domains (**Supplementary Figure 5**).

**Figure 3:**
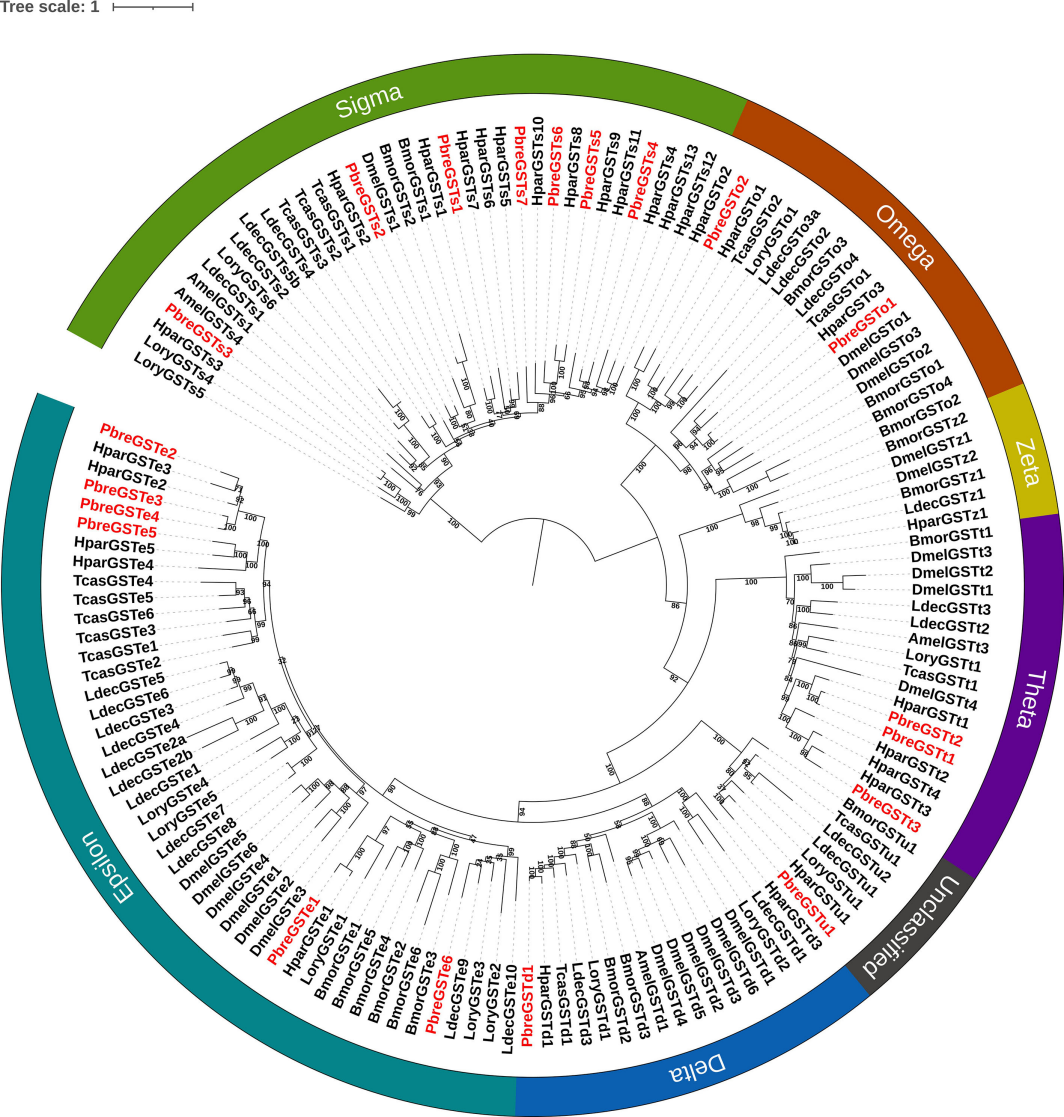
Phylogenetic analysis of GSTs from *P. brevitarsis* (Pbre) and other insects including *A. mellifera* (Amel), *B. mori* (Bmor), *D. melanogaster* (Dmel), *T. castaneum* (Tcas), *H. parallela* (Hpar), *L. oryzophilus* (Lory), and *L. decemlineata* (Ldec). Accession numbers of the sequences or references to their sources are listed in **Supplementary Table 1**. The PbreGSTs are highlighted in red.

Phylogenetic analysis of cytosolic GSTs revealed their classification into six clades (excluding zeta) (**Figure 3**). The sigma clade contained the highest number of PbreGSTs. In the insect-specific delta and epsilon clades, a total of 7 PbreGSTs were identified, with their quantity roughly equivalent to the number reported by Liu et al. in the *H. parallela* antennal transcriptome (28) (**Figure 3**). In addition, the omega, theta, and unclassified clades contained 2, 3, and 1 members of PbreGSTs respectively (**Figure 3**). These cytosolic *GSTs* displayed significant differential expression in the antennae, spanning an FPKM range of 0.89 to 978.44 (**Supplementary Table 3**). Among them, six *PbreGSTs* with higher FPKM values were classified into the delta, epsilon, and sigma clades (**Supplementary Table 3**).

### 3.5 Identification of *UGTs*

Ten *PbreUGTs* were annotated, and then named by the UGT Nomenclature Committee as *PbreUGT324BY1*-*PbreUGT312G4* (**Supplementary Table 3**). Among these, 6 *PbreUGTs* had intact ORFs encoding 509–525 amino acids (**Supplementary Table 3**). Signal peptides were predicted in 9 PbreUGTs with intact N-terminus, and transmembrane domains were predicted in 8 PbreUGTs at their C-terminus (**Supplementary Table 3**). Blastx alignment indicated that PbreUGT50D19 had 82% sequence identity to the *H. oblita* UGT, whereas the other PbreUGTs ranged from 47% to 67% (**Supplementary Table 3**). The C-terminal structure of these PbreUGTs were highly conserved, particularly two sugar donor-binding regions (**Supplementary Figure 6**). In these two regions, most PbreUGTs (9) contained eight key amino acid residues that interact with sugar donorsare (**Supplementary Figure 6**).

**Figure 4:**
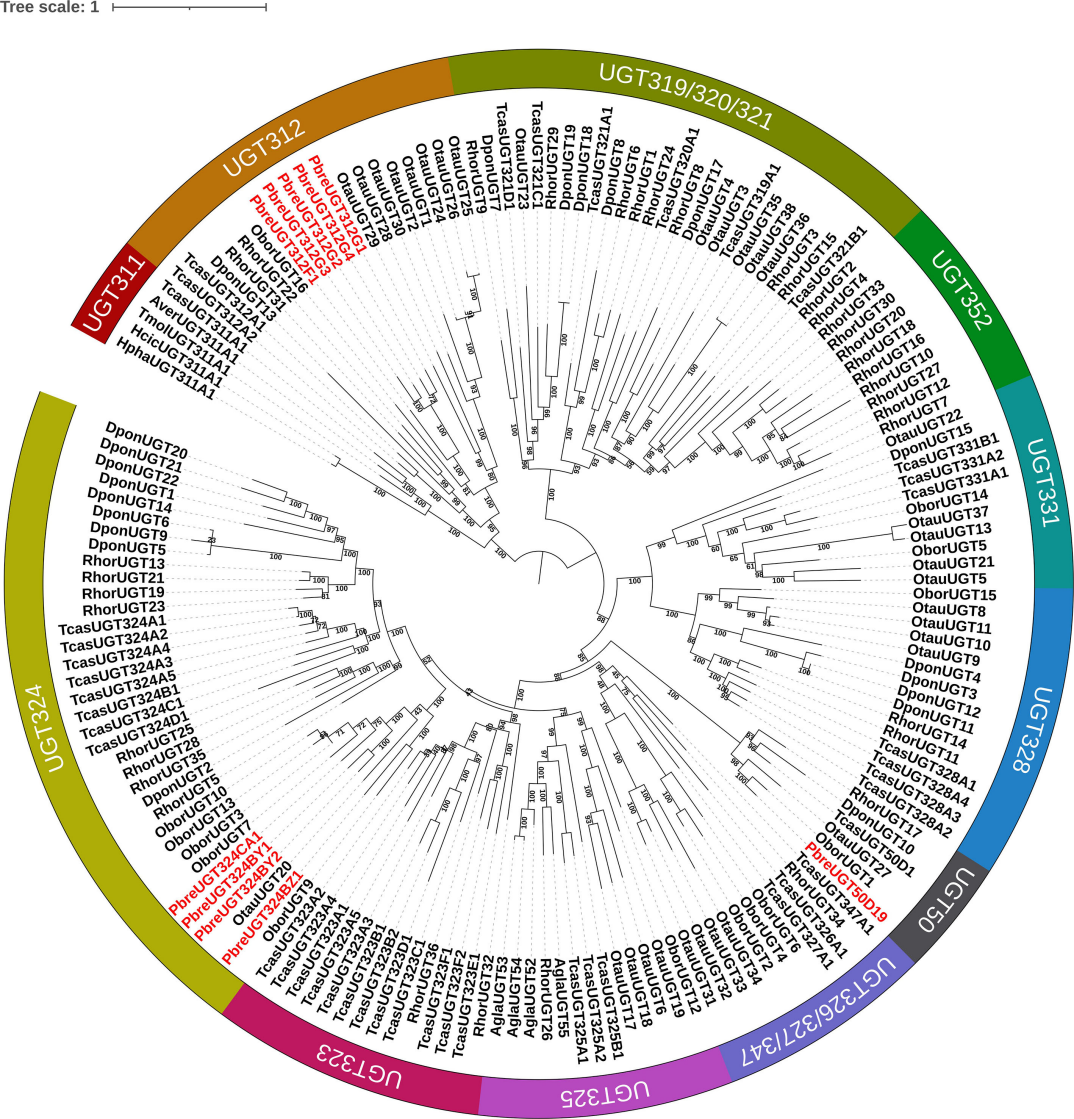
Phylogenetic analysis of UGTs from *P. brevitarsis* (Pbre) and other insects including *T. castaneum* (Tcas), *D. ponderosae* (Dpon), *R. horsfieldi* (Rhor), *O. taurus* (Otau), *O. borbonicus* (Obor), *Asbolus verrucosus* (Aver), *Hycleus cichorii* (Hcic), *Hycleus phaleratus* (Hpha), *T. molitor* (Tmol), and *Anoplophora glabripennis* (Agla). Accession numbers of the sequences or references to their sources are listed in **Supplementary Table 1**. The PbreUGTs are highlighted in red.

In the phylogenetic tree constructed using UGTs from Coleoptera (**Figure 4**), lineage-specific amplification was evident in clades like UGT324 and UGT312, which comprised 4 and 5 members in *P. brevitarsis*, respectively (**Figure 4**). Meanwhile, as seen in other species, the highly conserved UGT50 is represented by a single member (**Figure 4**). Quantitative analysis identified two genes with high expression in the antennae: *PbreUGT312F1* and *PbreUGT324BY2* (**Supplementary Table 3**).

### 3.6 Tissue expression profiles of 17 genes with High FPKM values in antennae

**Figure 5:**
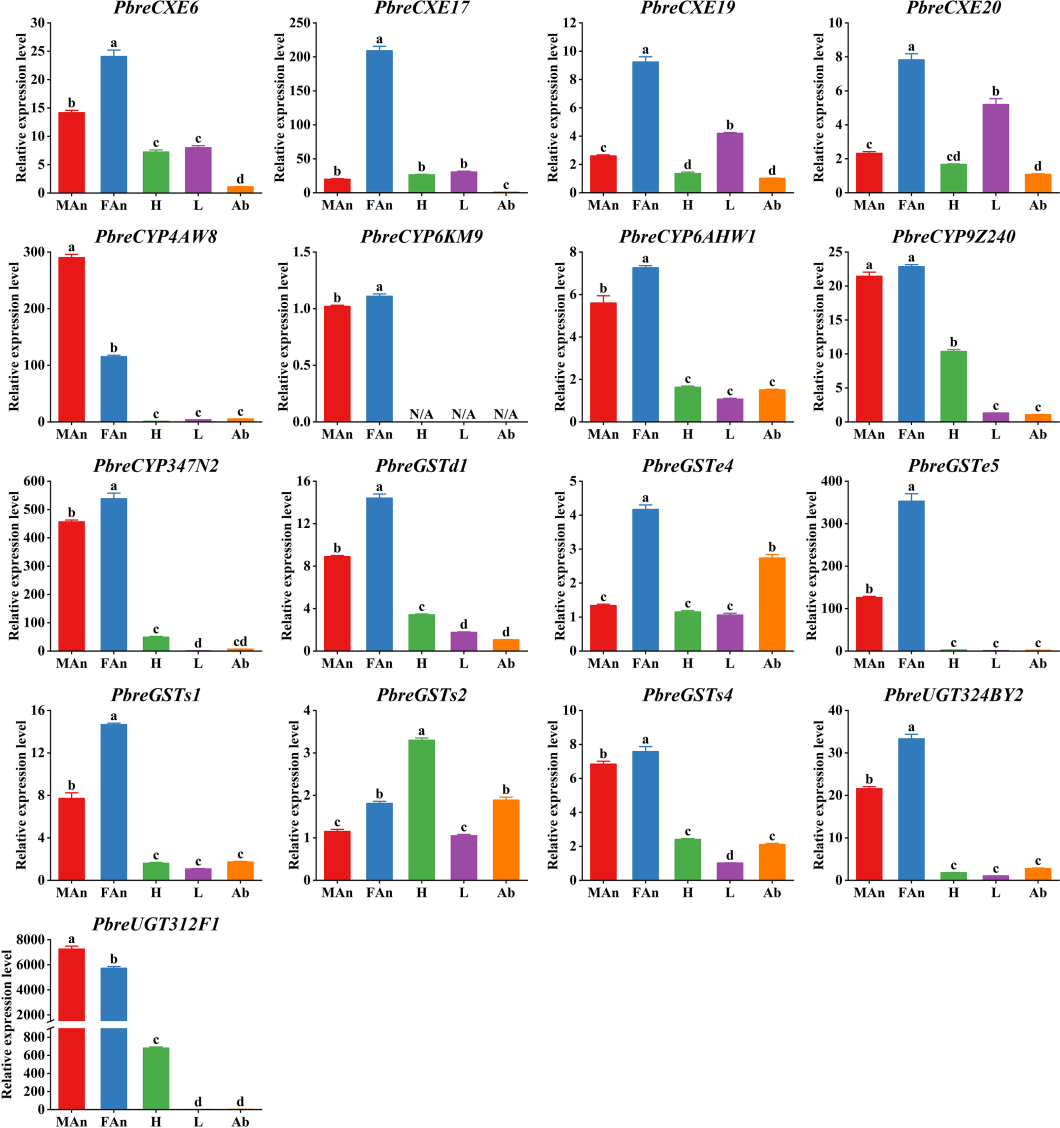
Relative expression levels of 17 genes in different adult tissues of *P. brevitarsis*. Tissue abbreviations: MAn, male antennae; FAn, female antennae; H, heads with antennae removed; L, legs; Ab, abdomens. Results are presented as the mean ± standard error (SE) of three technical replicates. Different lowercase letters indicate significant differences among tissues (one-way ANOVA with Tukey’s test, *p* < 0.05). N/A indicates that the transcript level was too low to be detected.

Although all 94 genes were functionally annotated, an FPKM >100 threshold was used to select high-abundance core candidates based on olfactory-predominant expression (8). The relative expression levels of these 17 genes (4 *CXEs*, 5 *CYPs*, 6 *GSTs*, and 2 *UGTs*) were then determined in various adult tissues. As shown in **Figure 5**, 16 of them exhibited significantly higher expression levels in male and/or female antennae compared to other tissues. Notably, *PbreCYP6KM9* displayed antennae-specific expression, with no detectable signal in the head, leg, or abdomen. Meanwhile, *PbreCXE19* and *PbreCXE20* were highly expressed in female antennae and leg, whereas *PbreCYP9Z24*0 localized primarily to the antennae of both sexes and the head. Additionally, *PbreGSTe4* showed the second-highest expression in the abdomen, surpassed only by that in female antennae (**Figure 5**). As the sole exception, *PbreGSTs2* exhibited peak expression in the head and ubiquitous distribution across all other tissues (**Figure 5**).

## 4 Discussion

Based on the antennal transcriptome from both sexes of *P. brevitarsi*s, this study systematically identified and analyzed enzyme families related to odor degradation, annotating a total of 94 genes, including 23 *CXEs*, 40 *CYPs*, 21 *GSTs*, and 10 *UGTs*. Currently, research on ODEs in Coleoptera remains relatively limited, with comprehensive annotation of candidate ODE genes conducted in only a few species. For instance, 20 *UGTs* (53), 20 *CXEs* (54), and 31 *GSTs* (28) have been successively reported in the antennae of *H. parallela*. Additionally, 134 and 155 odorant/xenobiotic degradation enzymes were identified in the antennal transcriptomes of *Agrilus planipennis* (55) and *D. valens* (56), respectively, while 246 candidate ODEs across four categories were identified in *Phyllotreta striolata* (57). Furthermore, existing studies have predominantly focused on cataloging a single ODE family within specific species (e.g., GSTs in *Lissorhoptrus oryzophilus* or *Sitophilus zeamais*) (27, 58). In this context, by simultaneously analyzing these multiple enzyme families, our study provides a more systematic and comprehensive perspective. More importantly, by integrating phylogenetic analysis with tissue expression profiling, we precisely screened for antennae-enriched expressed candidate genes. This work not only further enriches the available data on odor degradation within Scarabaeidae, but also provides a reliable molecular foundation for developing green pest control targets based on the olfactory behavior of *P. brevitarsis*.

Among the 23 CXEs of *P. brevitarsis*, PbreCXE1-PbreCXE20 possessed a complete catalytic triad structure, indicating their catalytic function. Further phylogenetic analysis revealed that these 20 CXEs were classified into two functional groups, forming five clades. PbreCXE1-PbreCXE8 belonged to the first functional group, which were responsible for the metabolic functions of general dietary or xenobiotic esters (11). Some moths (17, 59, 60) and *Blattella germanica* (61) have been found to possess odor degradation capabilities among members of this functional group. The second group was involved in the metabolism of hormones or pheromones (11). Among them, PbreCXE9-PbreCXE14 belonged to the “β-esterases and pheromone esterases” clade, which were clustered with PjapPDE in the Japanese beetle, known for their high-efficiency degradation activity of sex pheromones (14). This clade also included DmelEst-6, which has been detected to be expressed in the third antennal segment of *Drosophila* and functions as a direct ODE for various food esters (62). PbreCXE15 belonged to juvenile hormone esterases, a class of enzymes named after their substrates and typically act as specific degraders of juvenile hormones (63). However, in *Drosophila*, DmJHE has been found to be active against multiple odor esters, suggesting its potential to recognize a broader range of substrates (64). Five additional CXEs (PbreCXE16-PbreCXE20) were classified as Cuticular/antennal esterases, which may play a role in clearing hydrophobic xenobiotics (including pheromones) that enter into integument (65). For instance, the *Grapholita molesta* CXE5 can metabolize the pheromone component (Z/E)-8-dodecenyl acetate (66). The domain characteristics of PbreCXE21-PbreCXE23 indicated the loss of their catalytic activity, and they were classified into two non-catalytic clades in the phylogenetic tree: “Neurotactin” and “Gliotactin”. Consequently, they were considered to participate in neural development as cell adhesion molecules (11, 67).

Phylogenetic analysis revealed a striking asymmetric expansion within the *P. brevitarsis* CYP repertoire. Consistent with *T. castaneum* (52), the CYP3 and CYP4 clans were significantly amplified, with the CYP6, CYP4, and CYP9 families alone accounting for over half of the total members. In insect evolution, such family-specific expansions typically imply recent gene duplication events driven by ecological pressures to cope with diverse xenobiotics. Although direct functional prediction for these expanded PbreCYPs is precluded by the lack of strictly orthologous genes with biochemical validation, their clustering within these notoriously detoxification-associated families provides a critical macro-evolutionary context, indicating that this amplification likely serves to enhance the species’ overall chemical defense capabilities. Furthermore, within this broadly expanded repertoire, a subset of *PbreCYPs* exhibited high abundance in the antennae. Drawing parallels from other insects, we can cautiously predict an olfactory role for these specific members; for instance, highly abundant CYP6 and CYP4 members in the antennae of other species have been implicated in the recognition processes of plant volatiles (68) or alarm pheromones (22). This suggests a nuanced evolutionary trajectory where, following massive duplication, certain copies were co-opted for specialized odorant degradation, while the broader clade likely retains general detoxification functions. Conversely, the highly conserved CYP2 and mitochondrial clans, which contain members crucial for endocrine regulation (e.g., ecdysteroid metabolism) (69–72) and cuticle formation (73), showed no such expansion, indicating their essential, conserved basal roles.

The GSTs in insects are classified into two major types: cytosolic and microsomal classes, with the former primarily involved in cellular detoxification (74). Twenty cytosolic GSTs were annotated in the antennae of *P. brevitarsis*. Currently, GST expression in antennae has been observed in species of the orders Diptera, Lepidoptera, Coleoptera, and Homoptera (24, 75). This expression is considered to protect olfactory organs from toxic xenobiotics (24, 76), and GSTs enriched in antennae may play specific roles in olfactory responses (26–28, 58). Several GST genes in *P. brevitarsis* (e.g., *PbreGSTd1*, *PbreGSTe4*, *PbreGSTe5*, *PbreGSTs1*, *PbreGSTs2*, and *PbreGSTs4*) showed extremely high FPKM values in the antennae. In particular, *PbreGSTd1* exhibited the highest transcript expression level in female antennae, with an FPKM value of 978.44. Providing phylogenetic context for this high expression, PbreGSTd1 clustered with the delta GSTs of *H. parallela* (28) and *L. oryzophilus* (58)—both of which have been functionally linked to olfactory processes—as a small branch with high bootstraps, suggesting a potential functional conservation within this clade.

Similarly, UGTs contribute to the inactivation and clearance of xenobiotics, including pheromones and plant volatiles, within the olfactory system (31, 34). In *P. brevitarsis*, two antennal *PbreUGTs* (*PbreUGT324BY2* and *PbreUGT312F1*) were highly expressed compared to other members, a pattern mirroring the highly expressed *GSTs* (e.g., *PbreGSTd1*), which underscores the active involvement of both families in xenobiotic clearance. Furthermore, this coexistence of highly and lowly expressed members is a consistent quantitative pattern across all four identified enzymatic families. For instance, the FPKM values of the 40 *CYPs* ranged dramatically from 0.72 to 920, with similarly substantial variation observed in *CXEs*, *GSTs*, and *UGTs*. Taken together with the structural and phylogenetic divergence previously observed, this broad heterogeneity in transcript abundance suggests that these expanded families may not simply retain widespread functional redundancy. Rather, it implies a degree of functional specialization, where highly expressed genes are likely specifically recruited to process high loads of specific odorants or xenobiotics, whereas lowly expressed members might maintain basal metabolic levels, act as genetic backups, or be activated only under particular physiological conditions.

To more accurately identify ODE candidates among these four gene families, transcript-level tissue expression profiling was performed on 17 genes based on previous analysis and the screening criterion of FPKM values >100. The results demonstrated that the majority of genes were enriched in male and/or female antennae, and one antennae-specific gene (*PbreCYP6KM9*) was identified. Previous studies have indicated that such specifically expressed genes are preferred targets for investigating odor degradation functions (20, 23). Except for *PbreCYP6KM9*, the genes enriched in antennae were also expressed in other tissues, though this does not conflict with their antennae-specific functions (8). Taking the diamondback moth (*Plutella xylostella*) as an example, the antennae-enriched *PxylCCE16c* also exhibited high expression levels in the abdomen and leg (77). And the purified recombinant PxylCCE16c was able to degrade two sex pheromone components (17).

Only one gene (*PbreGSTs2*) displayed peak expression in the head, with considerable expression levels also observed in the other four tissues. Although the head used in our experiment was antennae-free, it still contained mouthpart appendages such as maxillary palps, resulting in many chemoreceptors on its surface. In *B. mori*, it has been reported that many CXEs were expressed in the antennae and maxilla, which are crucial olfactory organs for larvae, and it was predicted that they would play important roles in degradation of volatile acetate allelochemicals and adaptive evolution of silkworms to mulberry leaves (65). Furthermore, *UGT46A1* in silkworms was highly expressed in the larval head and shown significantly higher expression levels in the adult stage compared to other developmental stages (35). RNA interference knockdown of this gene altered the feeding habits of silkworms, suggesting that UGT46A1 may regulate feeding behavior by influencing the olfactory system (35). In light of these analogous examples, the potential olfactory function of PbreGSTs2 cannot be ruled out.

Despite these findings, certain limitations should be acknowledged. The identification of ODE candidates relied primarily on transcriptomic and expression profiling. While antennal-enrichment provides strong correlative evidence for their olfactory roles, direct functional validation was not conducted in this phase. Consequently, the precise substrate specificities and physiological functions of these candidates remain to be elucidated. Therefore, our current results serve as a foundational screening to prioritize targets. Given that specific plant volatiles have been reported to possess attractant activity toward *P. brevitarsis* (78), and the aggregation pheromone components of this species have been identified (79), these compounds serve as highly relevant test compounds. Subsequent computational validations utilizing molecular docking and molecular dynamics (MD) simulations could be applied to assess the binding stability and conformational dynamics between these ODEs and these potential ligands (80). More importantly, building on frameworks that integrate such *in silico* predictions with subsequent behavioral assays (81), future research will be required to definitively confirm ODE functions. Practically, these validated ODEs could serve as novel targets for developing inhibitors that disrupt insect olfaction, or guide the rational design of structurally modified semiochemicals to extend the field longevity of lures. Ultimately, translating this molecular understanding into practice will facilitate the development of highly effective, olfaction-based pest management strategies.

## 5 Conclusions

In summary, this study systematically identified and analyzed the olfactory-related enzymes in *P. brevitarsis* through antennal transcriptome sequencing, bioinformatics analysis, and tissue expression profiling, annotating a total of 94 genes across four families. Among these, 16 genes exhibited enriched or specifically high expression in antennae. We propose these genes as primary candidates for ODEs, serving as a prioritized list for future functional validation. Our work lays the foundation for elucidating the odor signal inactivation mechanism in *P. brevitarsis*, expands the transcriptomic resource of ODEs in Scarabaeidae, and provides a theoretical basis for developing novel pest management targets based on olfactory behavior.

## Data Availability

The datasets presented in this study can be found in online repositories. The names of the repository/repositories and accession number(s) can be found in the article/**Supplementary Material**.
