# Supplementary material for: Odorant degrading enzyme candidates enriched in the antennae of the white-spotted flower chafer, Protaetia brevitarsis
Source: Front Insect Sci. 2026 Jun 3;6:1843786. doi: 10.3389/finsc.2026.1843786 (PMC13272398; doi:10.3389/finsc.2026.1843786)
Supplement: Supplementary file 1 [file Supplementaryfile1.docx]

Supplementary Material


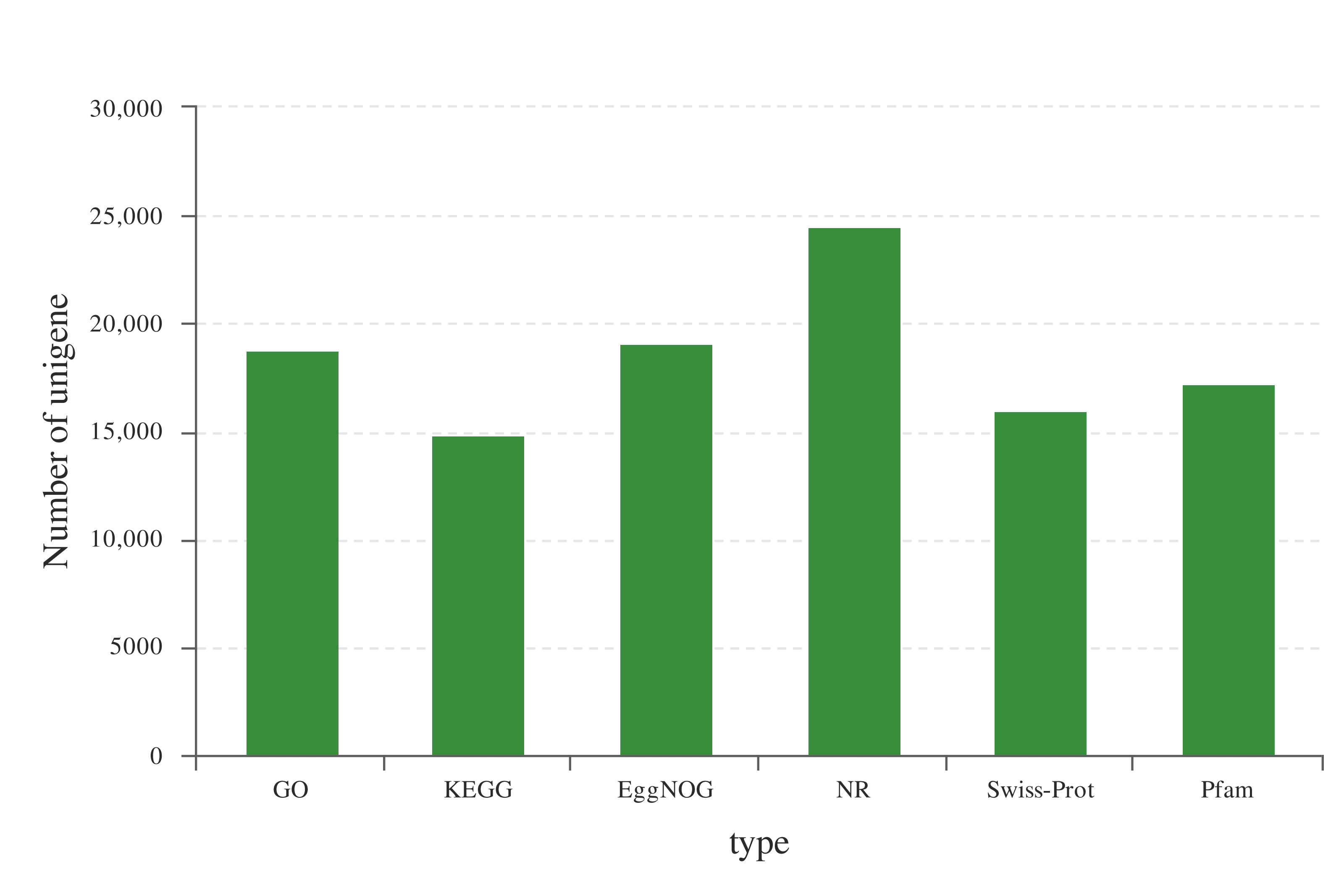

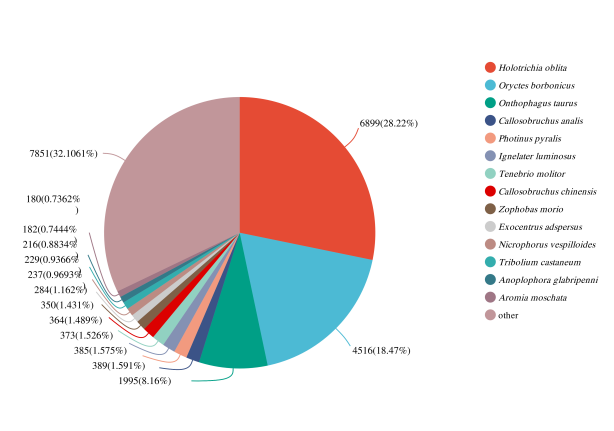


A

B


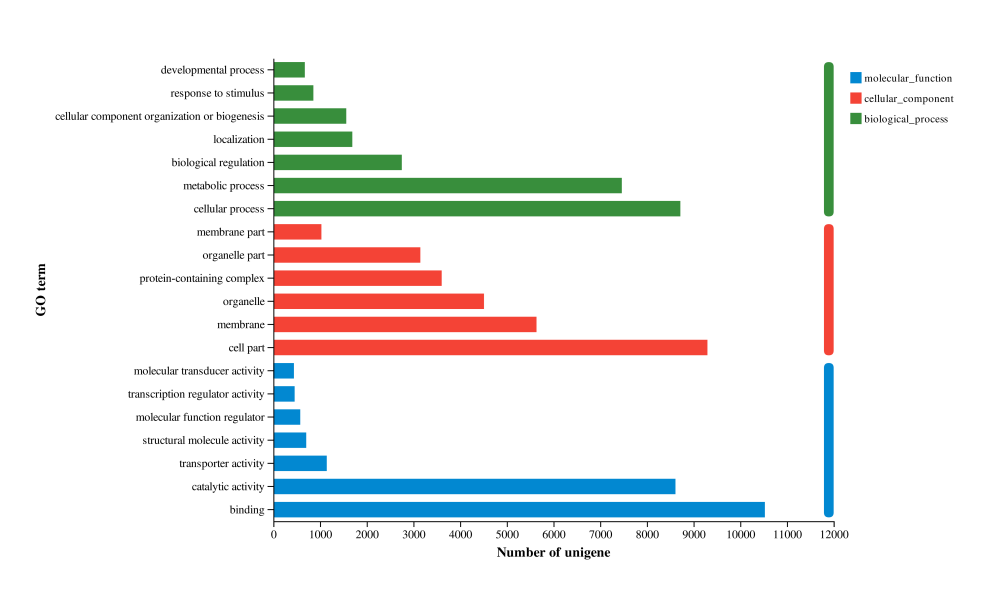


D

C


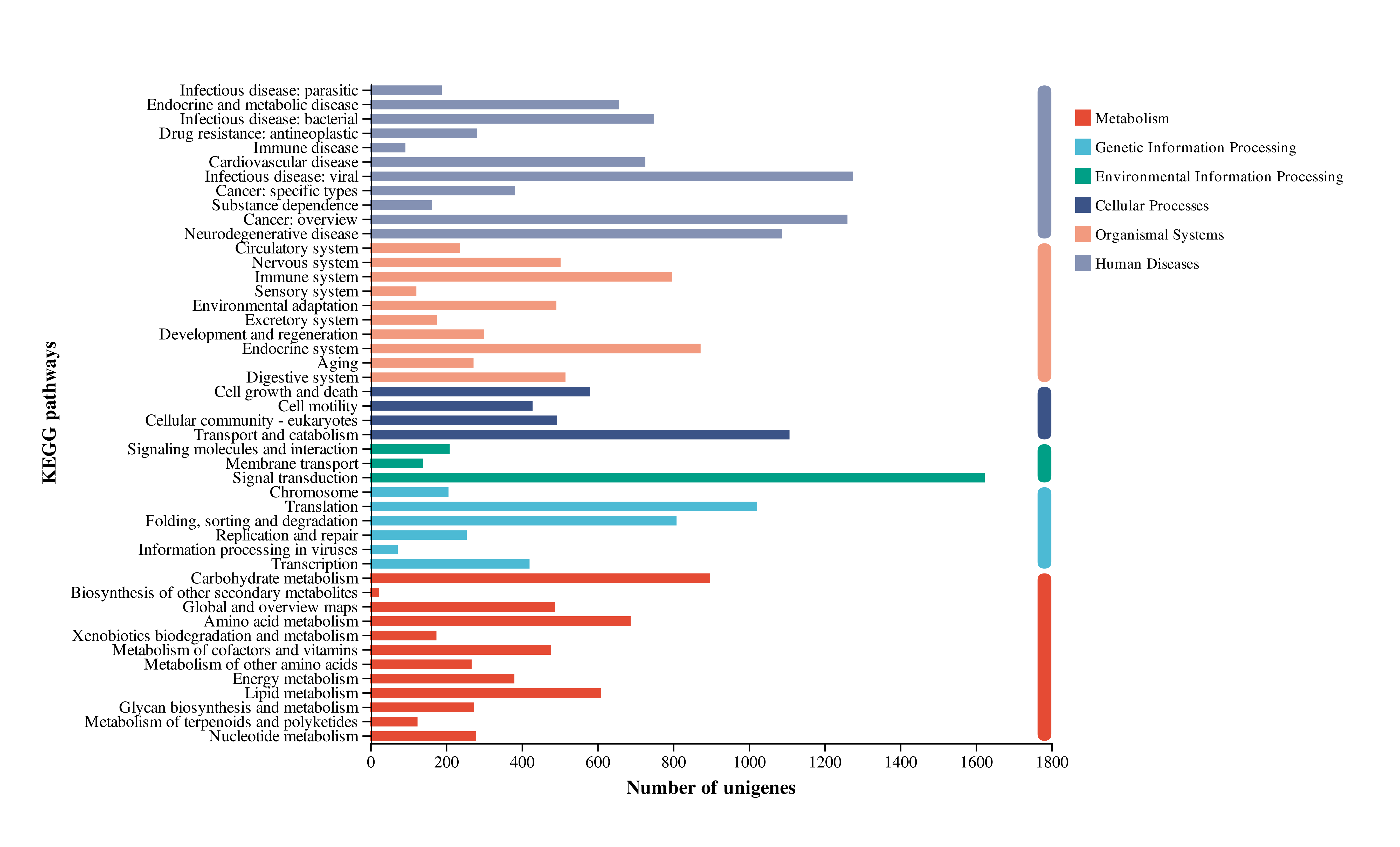


**Supplementary Figure 1.** Functional annotation of unigenes. (A) Number of unigenes with functional annotations in six databases. (B) Species distribution based on NR database alignment. (C) GO annotations analysis (Level2). (D) KEGG annotations analysis.


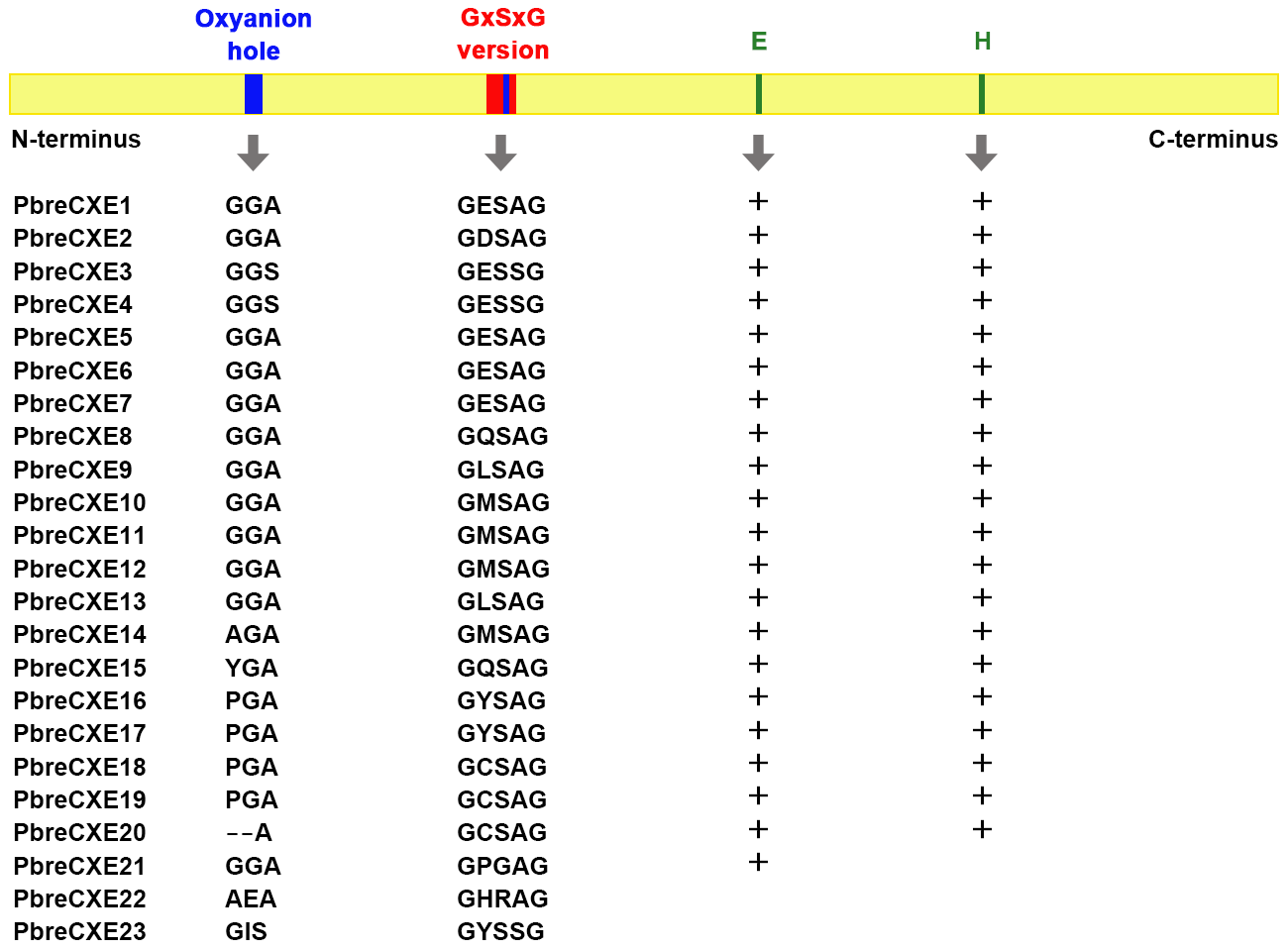


**Supplementary Figure 2.** Catalytic motifs of the *P. brevitarsis* CXEs. The dashed line indicates the incomplete N-terminus of PbreCXE20.


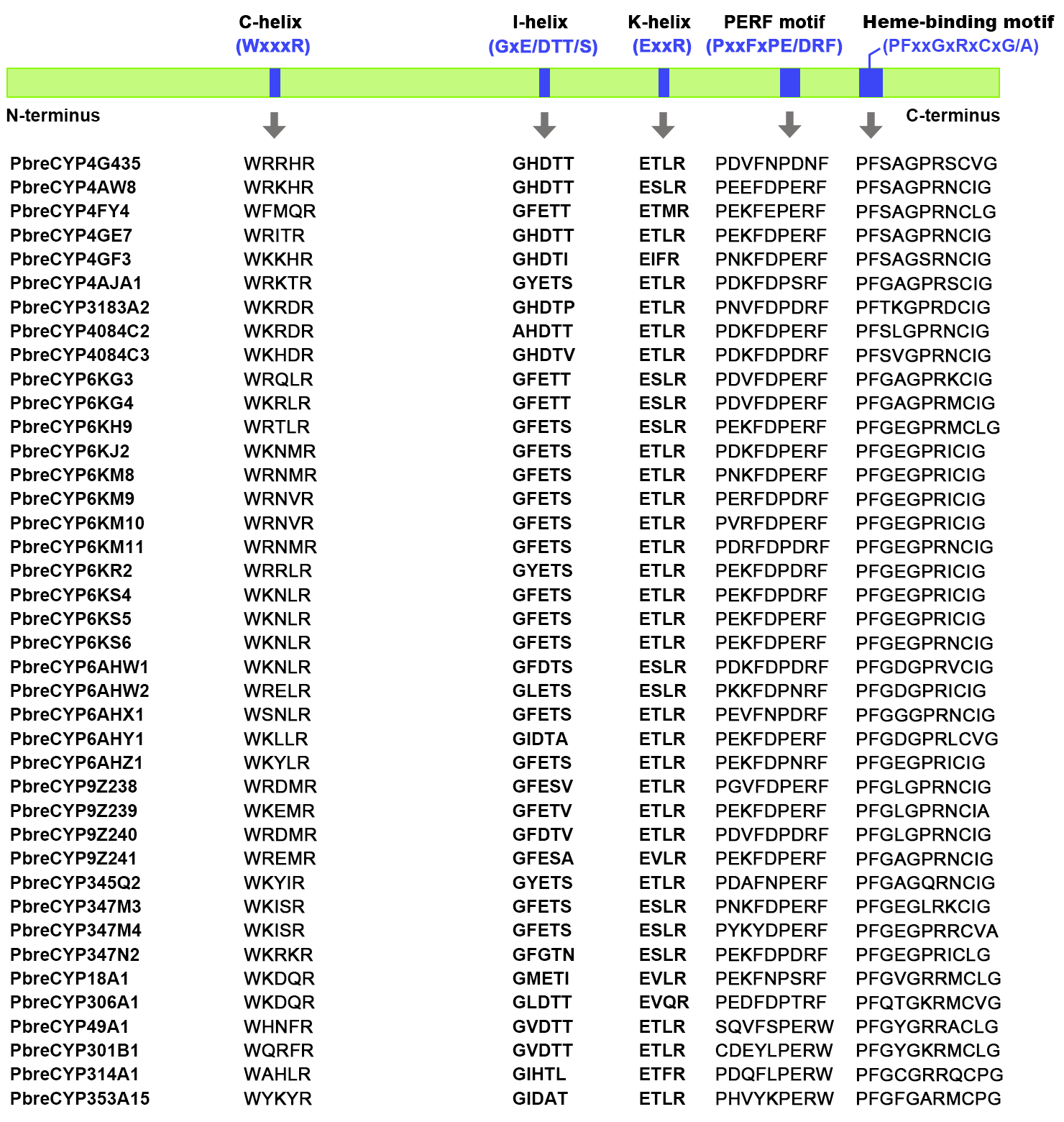


**Supplementary Figure 3.** Conserved domains of the *P. brevitarsis* CYPs.


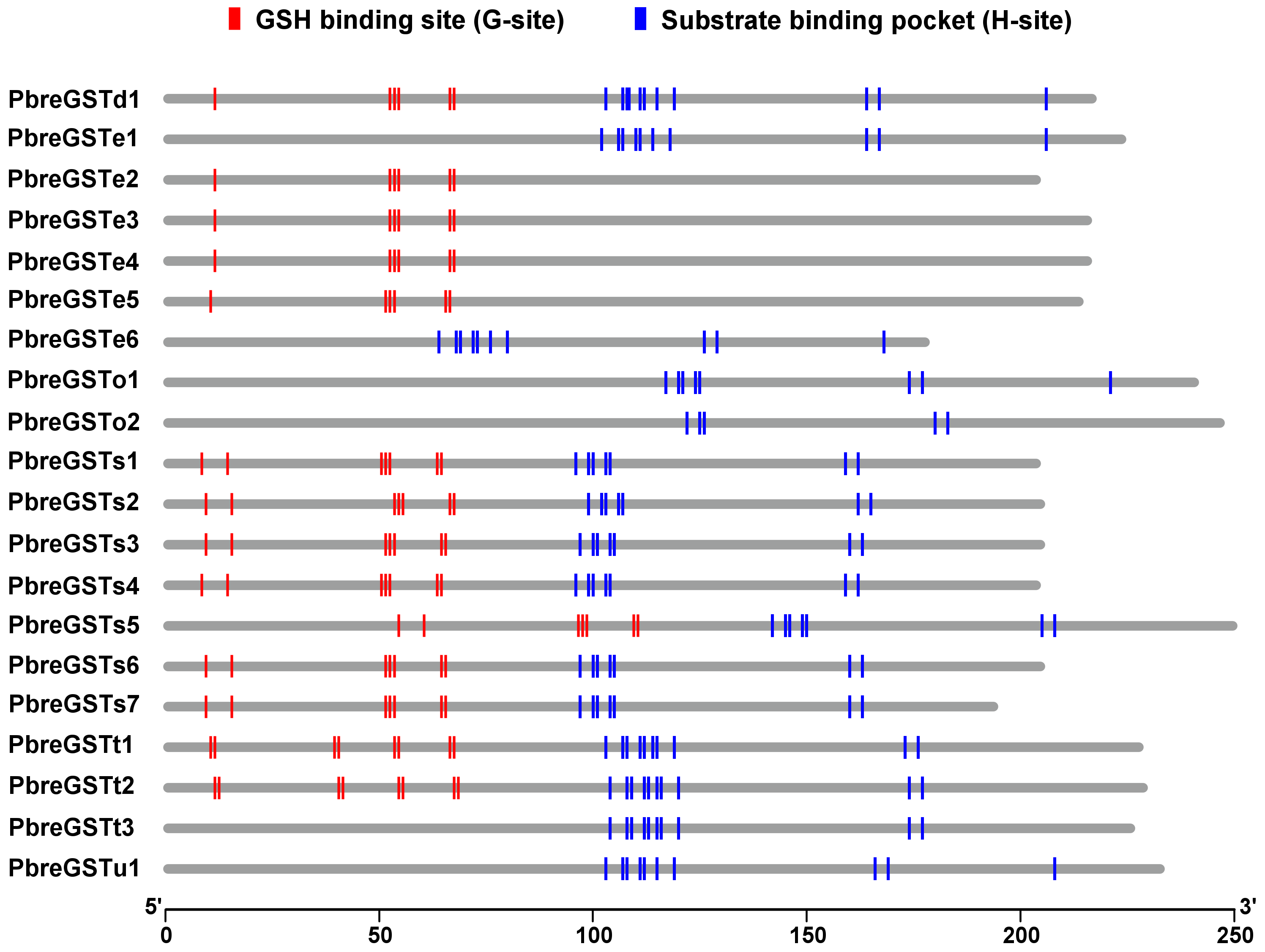


**Supplementary Figure 4.** Predicted GSH binding site (G-sites) and substrate binding pocket (H-sites) of the *P. brevitarsis* cytosolic GSTs.


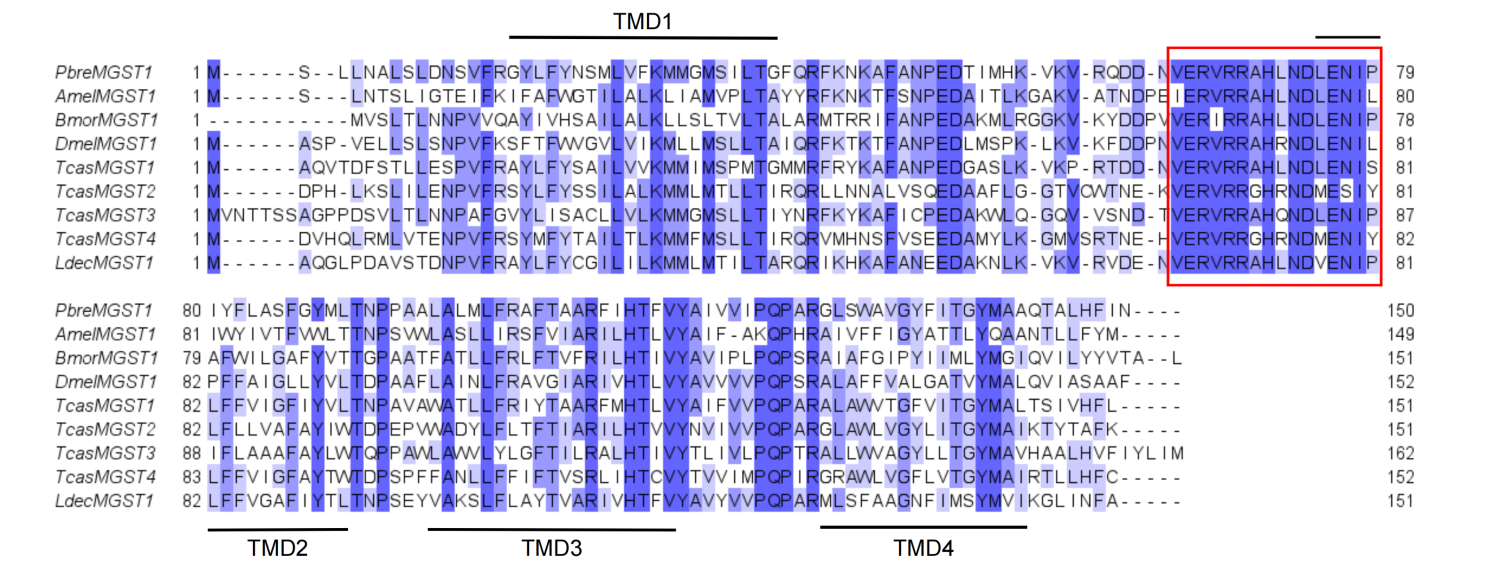


**Supplementary Figure 5.** Multiple alignments of microsomal GSTs from six insects. TMD indicates transmembrane domains. Conservative motif is highlighted in red box.


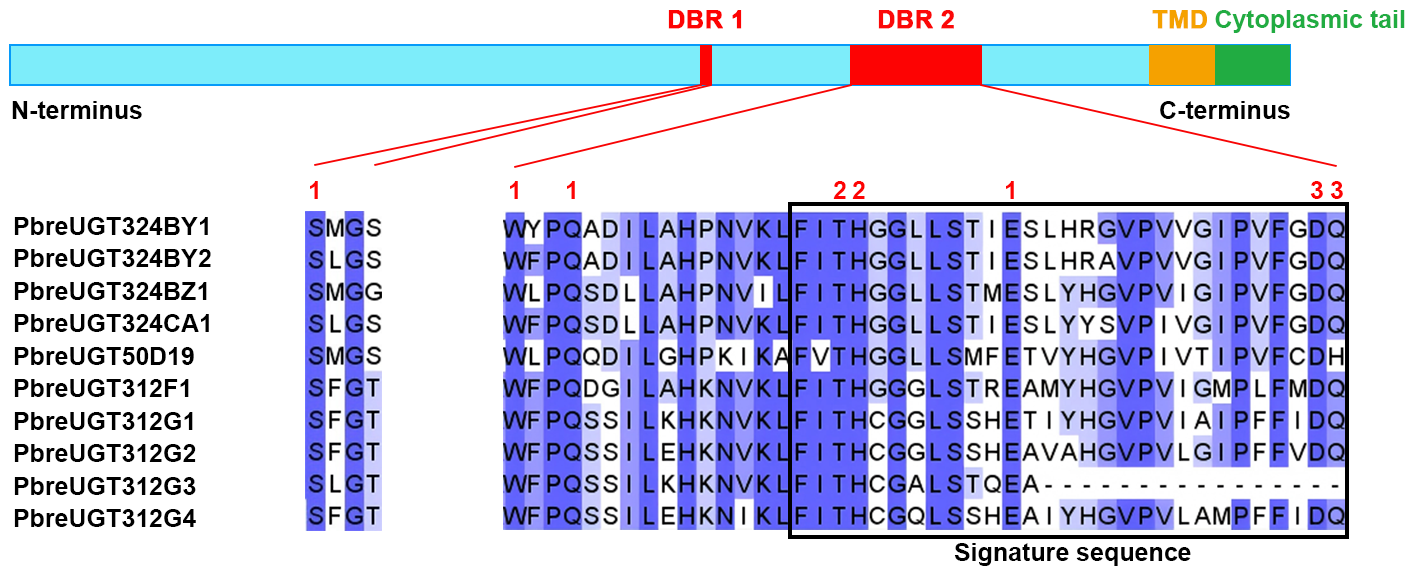


**Supplementary Figure 6.** Multiple alignments of the *P. brevitarsis* UGTs. The upper panel illustrates the conserved C-terminal structure of UGTs, including two sugar donor-binding regions (DBR 1 and DBR 2, red), a transmembrane domain (orange), and a cytoplasmic tail (green). The lower panel shows the alignments of the DBR 1 and DBR 2 regions. Key amino acid residues interacting with sugar donorsare labeled with red numbers, including nucleotide interaction residues (1), phosphate interaction residues (2), and glucoside interaction residues (3). The dashed line indicates the incomplete C-terminus of PbreUGT312G3.
